# Angular-momentum nanometrology in an ultrathin plasmonic topological insulator film

**DOI:** 10.1038/s41467-018-06952-1

**Published:** 2018-10-24

**Authors:** Zengji Yue, Haoran Ren, Shibiao Wei, Jiao Lin, Min Gu

**Affiliations:** 1 Laboratory of Artificial-Intelligence Nanophotonics, School of Science, RMIT University, Melbourne, Victoria 3001 Australia; 2 Centre for Ultrahigh-bandwidth Devices for Optical Systems (CUDOS), School of Science, RMIT University, Melbourne, Victoria 3001 Australia; 3 School of Engineering, RMIT University, Melbourne, Victoria 3001 Australia

## Abstract

Complementary metal–oxide–semiconductor (CMOS) technology has provided a highly sensitive detection platform for high-resolution optical imaging, sensing and metrology. Although the detection of optical beams carrying angular momentum have been explored with nanophotonic methods, the metrology of optical angular momentum has been limited to bulk optics. We demonstrate angular-momentum nanometrology through the spatial displacement engineering of plasmonic angular momentum modes in a CMOS-compatible plasmonic topological insulator material. The generation and propagation of surface plasmon polaritons on the surface of an ultrathin topological insulator Sb_2_Te_3_ film with a thickness of 100 nm is confirmed, exhibiting plasmonic figures of merit superior to noble metal plasmonics in the ultraviolet-visible frequency range. Angular-momentum nanometrology with a low crosstalk of less than −20 dB is achieved. This compact high-precision angular-momentum nanometrology opens an unprecedented opportunity for on-chip manipulation of optical angular momentum for high-capacity information processing, ultrasensitive molecular sensing, and ultracompact multi-functional optoelectronic devices.

## Abstract (web summary)

Multiplexed vortex light beams are promising for optical communications, but efficient mode sorting is so far limited to bulk optics. Here the authors develop a scalable vortex beam sorter that uses a plasmonic topological insulator structure to spatially separate the modes to resolve them on a standard CMOS detector.

## Introduction

Optical imaging, sensing and metrology have been significantly advanced with the development of complementary metal–oxide–semiconductor (CMOS) technology^1–4^. Its unique capability to support photonic devices with high-spatial and -spectral resolution has allowed the detection of optical signals in different physical dimensions. The orbital angular momentum (OAM) of light carried by a helical wavefront with a physically unbounded set of spatial modes has emerged as a physically orthogonal dimension of light^5^. Although the generation and measurement of the OAM of light have recently been exploited by nanophotonic methods^6–10^, the separation of OAM modes has been limited to bulk optics that accomplish the linear displacement of OAM-carrying beams^11,12^, due to the lack of OAM-dispersive materials or detectors at a nanoscale.

Plasmonics has merged high-capacity photonics and electronics at nanoscale dimensions^13,14^. As such, nanoplasmonic manipulation and multiplexing of the OAM of light have recently been explored for widespread applications ranging from on-chip optical signal processing^15^ and high-definition displays^16,17^ to high-precision optical micromanipulation^18^ and highly sensitive chiral molecular detection^19^. However, observation of linearly OAM-dispersive plasmonic structures is restricted to the characterization by a near-field scanning optical microscopy^20^, due to the absence of an outcoupling mechanism for the transmission of surface plasmons to far-field detectors, which is essential for on-chip OAM nanometrology devices.

On the other hand, topological insulator materials, with a three-dimensional high-index insulating bulk state and a topologically protected two-dimensional metallic surface state, have recently been shown to support localized surface plasmon resonances in an ultrabroad frequency band ranging from the ultraviolet to terahertz frequencies^21–23^. Such an ultrabroad operation bandwidth of plasmonics in CMOS-compatible topological insulator materials is highly desirable for integrated photonic detectors, although it is generally not available in conventional noble metals^24–26^. To date, however, the surface plasmon polaritons (SPPs) have not been experimentally achieved in topological insulator materials at visible frequencies.

Here, we demonstrate a concept of CMOS-integratable OAM nanometrology in an ultrathin topological insulator film, based on the linear displacement engineering of plasmonic angular momentum fields at the nanoscale. Our dispersion analysis suggests that the SPPs in the topological insulator Sb_2_Te_3_ thin film exhibit superior plasmonic figures of merit as compared to the noble metal gold in the ultraviolet-visible spectral region. Applying the superior plasmonic effect in an ultrathin Sb_2_Te_3_ film with a thickness of 100 nm to the linear displacement engineering of OAM-carrying plasmonic modes at the nanoscale, we demonstrate for the first time a CMOS-integratable OAM nanometrology device with a low modal crosstalk of < −20 dB. Our demonstrated ultrathin OAM nanometrology device with a small footprint of 13.4 µm by 10.6 µm may open exciting avenues for the future development of ultrathin functional optoelectronics.

## Results

### Design of OAM nanometrology in an ultrathin Sb_2_Te_3_ film

**Fig. 1:**
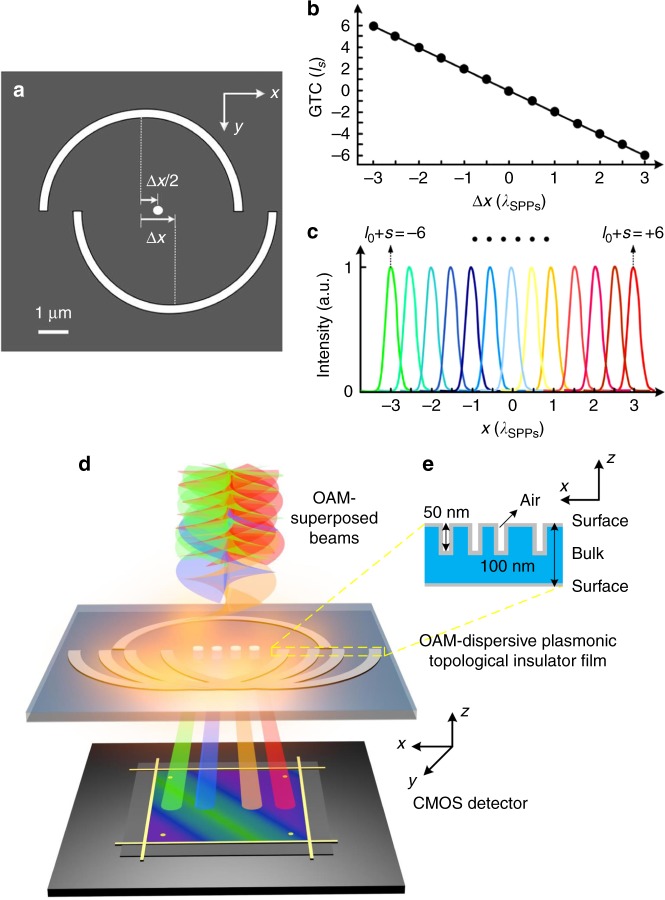
Principle of OAM nanometrology in a plasmonic topological insulator film. **a** The schematic design of different GTCs by shifting one half of a circular nanogroove with respect to the other along the breaking line. The GTCs are inspected in the middle of the transverse shifts (∆*x*/2), where ∆*x* is the shifting distance. **b** The linear dependence of the GTC (*l*_S_) on the transverse shift (∆*x*) of the two semi-circular nanogrooves. *λ*_SPPs_ represents the SPPs wavelength. **c** The intensity cross-sections in the transverse shift direction (the *x*-axis) of plasmonic fields excited by illuminating optical beams with incident angular momentum modes (*l*_0_ + *s*) ranging from −6 to +6 on displaced semi-circular nanogrooves with the GTCs (*l*_S_) ranging from +6 to −6, respectively. **d** The schematic of CMOS-integratable OAM nanometrology. An ultrathin OAM-dispersive plasmonic topological insulator film is constituted by spatially shifted semi-circular nanogrooves and mode-sorting nanoapertures, through which the OAM-superposed beams are spatially separated and directly measured by a CMOS detector in the far-field region. **e** The multilayer structure of the topological insulator thin film as well as the cross-section of nanogrooves

The design principle and the schematic of CMOS-integratable OAM nanometrology are illustrated in Fig. 1. Owing to the diffractive propagation of the SPPs, Archimedean spirals^27,28^ and shifted semi-circular nanostructures^17^ were demonstrated to transfer their geometry-dependent geometrical topological charges (GTCs, *l*_S_) to plasmonic angular momentum modes. As such, an incident optical beam with the spin angular momentum, SAM (*s*), and the OAM (*l*_0_) can be converted to a plasmonic angular momentum field with the total angular momentum (*L*) mode of *L* *=* *s* *+* *l*_0_ *+* *l*_S_, governed by the angular momentum conservation in the SPPs coupling. A key enabling aspect of the OAM nanometrology is to design plasmonic nanostructures with different GTCs (*l*_S_) to couple optical beams with different angular momentum modes (*l*_0_ + *s*) into linearly displaced plasmonic fields. Here we use spatially shifted semi-circular nanogrooves to achieve a linear spatial displacement of plasmonic angular momentum modes. Owing to the geometric phase originated from the spin–orbit coupling in the SPPs excitation, unidirectional shifting one half of a circular nanogroove with respect to the other half along the breaking line (Fig. 1a) allows the continuous generation of GTCs, *l*_S_, at the middle locations of the spatial shifts (Fig. 1b). As an example, we numerically simulated the generation of a range of GTCs from −6 to 6 by shifting the semi-circular nanogroove from 3*λ*_SPPs_ to −3*λ*_SPPs_, respectively, where *λ*_SPPs_ represents the SPPs wavelength (Supplementary Fig. 1). As a result, the semi-circular nanogrooves shifted by different amounts (correspond to the GTC of *l*_S_) can be utilized to convert optical beams with different SAM and OAM modes (*s* + *l*_0_ = −*l*_S_) into a solid plasmonic focus at the middle location of the displaced semi-circular nanogrooves, which is governed by the total angular momentum conservation (*L* = *s* + *l*_0_ + *l*_S_ = 0). Figure 1c shows the simulated intensity cross-section of plasmonic fields excited by illuminating optical beams with different angular momentum modes (*s* + *l*_0_) ranging from −6 to 6 on semi-circular nanogrooves with shift-dependent GTCs (*l*_S_) ranging from +6 to −6, respectively, which reveals the linear displacement of angular momentum modes at the nanoscale. To directly measure the angular momentum-dependent spatial shift in the far-field region, angular momentum mode-sorting nanoapertures are allocated in the middle of the spatial shifts to couple out plasmonic angular momentum fields, laying the physical foundation for OAM nanometrology.

The schematic design of CMOS-integratable OAM nanometrology is given in Fig. 1d. A set of OAM-superposed beams are normally incident on an ultrathin OAM-dispersive plasmonic topological insulator film with a thickness of 100 nm. Through the interaction with shallow semi-circular nanogrooves with a depth of 50 nm (inset of Fig. 1d), the incident OAM beams are converted into spatially separated SPPs fields confined on the surface of the Sb_2_Te_3_ thin film. Subsequently, the SPPs fields are coupled out by mode-sorting circular nanoapertures drilled through the thin film, leading to OAM-dependent linear spatial displacement in the detection plane of a CMOS camera (THORLABS, DCC1645C).

### Dispersion analysis of the SPPs in a Sb_2_Te_3_ thin film

**Fig. 2:**
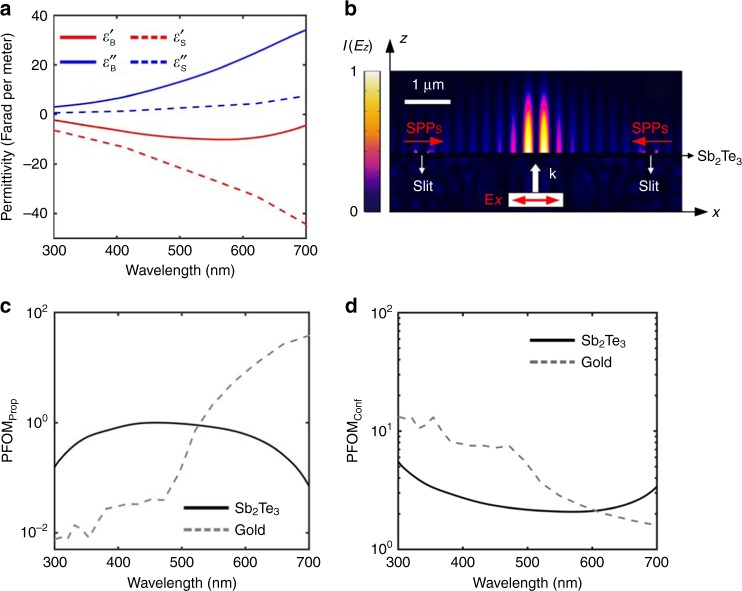
Dispersion analysis and figure of merit of the topological insulator thin film. **a** Real (red colour) and imaginary (blue colour) parts of the permittivity spectra of the bulk (solid curves) and surface (dashed curves) states of the Sb_2_Te_3_ thin film, respectively. The thickness of the bulk and the surface layer of Sb_2_Te_3_ thin film is 100 and 1.5 nm, respectively. **b** The numerically simulated normalized intensity distribution of the SPPs field excited at the interface between the Sb_2_Te_3_ thin film and the air. The thickness of the Sb_2_Te_3_ film is 100 nm. The width of air slits is 250 nm. The *x*-linearly polarized incident light has a wavelength of 640 nm. The period of the interference fringes of evanescent SPPs standing waves is 302 nm, which suggests the SPP wavelength of 604 nm in the numerical simulation. **c**, **d** Dependence of plasmonic figure of merit for propagation length (**c**) and degree of confinement (**d**) on the ultraviolet-visible wavelength for the Sb_2_Te_3_ thin film and the gold material, respectively

To perform the dispersion analysis of the SPPs in the topological insulator material of Sb_2_Te_3_, we experimentally measured the optical constants of the Sb_2_Te_3_ material using the ellipsometry method over a broad wavelength range from 300 to 700 nm. The conductive surface layer of the Sb_2_Te_3_ thin film features a thickness of 1.5 nm which was obtained from an ellipsometry fitting model. Specifically, B-spline and Tauc–Lorenz models were used to determine the permittivity of the surface and the bulk layer of the Sb_2_Te_3_ thin film, respectively^22,23,29^. The spectra of the real part of the permittivity of both the surface state and the bulk state of the Sb_2_Te_3_ thin film are shown in Fig. 2a. The real part of the permittivity of both the surface state and bulk state of the Sb_2_Te_3_ thin film remains negative in the whole measured wavelength range, which indicates that both the bulk state and surface state of the topological insulator Sb_2_Te_3_ can support the SPPs in principle. Moreover, the bulk dielectric permittivity of the Sb_2_Te_3_ thin film implies a large refractive index at optical frequencies, which might result from the complex electronic structures of topological insulator materials, including indirect gap, the density of state near the Fermi surface, and electron–hole interactions^30,31^. Figure 2b shows the electric field distribution of the SPPs at the interface between the Sb_2_Te_3_ thin film and air, which is excited at a visible wavelength of 640 nm by using a nanograting coupler.

To compare the plasmonic effect of the topological insulator thin film with that of noble metals, plasmonic figures of merit of the SPPs in terms of the two fundamental aspects of propagation length (PFOM_prop_) and degree of confinement (PFOM_conf_) are characterized^26^. The plasmonic figures of merit for the propagation length and the degree of confinement are defined as ${\mathrm{PFOM}}_{{\mathrm{prop}}} = L_{{\mathrm{SPP}}}/\lambda _{{\mathrm{SPP}}} = {{\mathrm{Re}}} (k_\parallel )/4\pi {{\mathrm{Im}}} (k_\parallel )$ and ${\mathrm{PFOM}}_{{\mathrm{conf}}} = \lambda _{\mathrm{0}}\,{\mathrm{Im}}(k_ \bot )$, respectively, where *k*_||_ and *k*_⊥_ are the transverse and longitudinal wave-vectors on the dielectric (air) side of the Sb_2_Te_3_/air interface, respectively, and *λ*_0_ is the excitation wavelength of light. To effectively evaluate the figures of merit of the Sb_2_Te_3_ thin film, its permittivity was experimentally retrieved from spectroscopic ellipsometry data, without modelling it as a multi-layered structure^22^ (Supplementary Fig. 2). By comparing with gold plasmonics, the Sb_2_Te_3_ thin film exhibits a much longer propagation length in a broad spectral region ranging from 300 to 500 nm (Fig. 2c) and a stronger SPPs confinement in the spectral range from 600 to 700 nm (Fig. 2d), respectively. The outperforming plasmonic effect in the Sb_2_Te_3_ thin film makes it a good candidate for a CMOS-compatible plasmonic material in the ultraviolet-visible spectral region. We further characterized the thickness-dependent reflectance and transmittance of the Sb_2_Te_3_ thin film (Supplementary Fig. 3a), which shows a flat reflectance curve with a value around 0.6 as well as a negligible transmittance when the thickness is above 50 nm. By fixing the thickness of the Sb_2_Te_3_ thin film to 100 nm, the reflectance and transmittance curves keep as a constant across the ultraviolet and visible spectra (Supplementary Fig. 3b).

### A subwavelength SPPs focus in an Sb_2_Te_3_ thin film

**Fig. 3:**
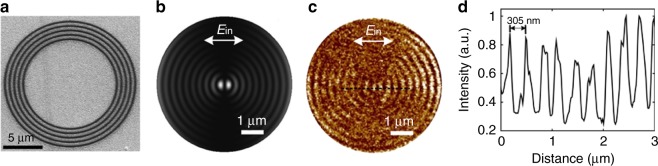
Characterization of a subwavelength SPPs focus in a Sb_2_Te_3_ thin film. **a** SEM image of the fabricated ultrathin plasmonic lens. **b** Simulated intensity distribution of the plasmonic focus excited by the plasmonic lens. The arrow shows the orientation of linearly polarized incident light. **c** Experimentally measured intensity distribution of the plasmonic focus by a SNOM system. **d** The cross-section of the measured intensity distribution of the plasmonic focus. The wavelength of the incident light beam is 640 nm. The period of the interference fringes of evanescent SPPs standing waves is around 305 nm which suggests the SPPs wavelength of 610 nm in the SNOM characterization

To experimentally confirm the generation and propagation of the SPPs in the surface of a topological insulator, we designed and fabricated a plasmonic lens consisting of five circular gratings with a period of 605 nm in a Sb_2_Te_3_ thin film with a thickness of 100 nm (Fig. 3a). Based on our numerical simulation, by normally illuminating a linearly polarized plane wave on concentric circular nanogrooves engraved in a Sb_2_Te_3_ thin film, a two-lobe SPPs focus originated from the interference of the SPPs fields on the nanogrooves can be excited (Fig. 3b). This SPPs focus was experimentally characterized by using a near-field scanning optical microscope (SNOM) at a visible wavelength of 640 nm (Fig. 3c). Through mapping the intensity distribution of the SPPs fields, a subwavelength SPPs focus with an effective wavelength of 610 nm is revealed (Fig. 3d). Moreover, the two-lobe intensity pattern of the SPPs focus changes its orientation when the incident linear polarization is switched to its orthogonal state (Supplementary Fig. 4).

### Characterization of the OAM nanometrology

**Fig. 4:**
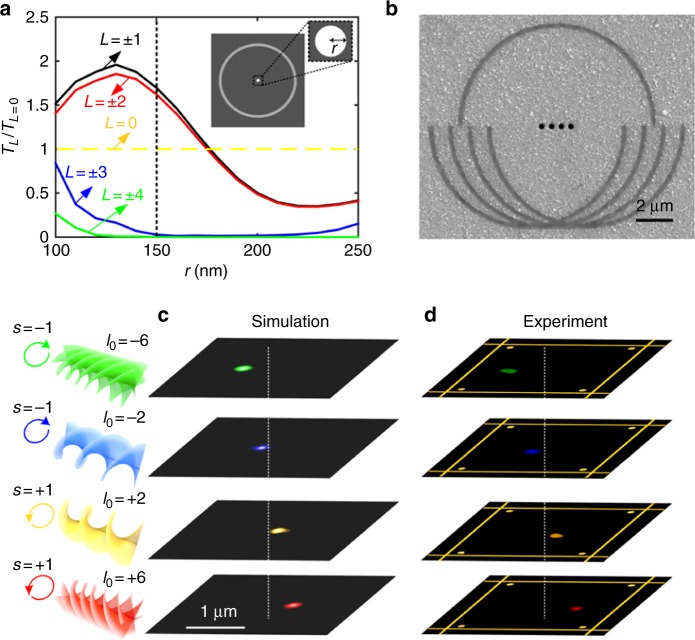
Characterization of OAM nanometrology in an ultrathin Sb_2_Te_3_ thin film. **a** Optical transmission of different total angular momentum modes *L* as a function of radius (*r*) of the mode-sorting nanoaperture. The transmittance curves for different angular momentum modes are normalized by the fundamental mode result with *L* = 0. The inset shows the schematic of the simulated nanostructures which consist of a shallow circular nanogroove and a drilled-through nanoaperture. The black dotted line labels out a mode-sorting nanoaperture waveguide with a radius of *r* = 150 nm used in the OAM nanometrology device. **b** SEM image of the fabricated four-states OAM nanometrology device. **c**, **d** Simulated (**c**) and experimental (**d**) characterization of the CMOS-integratable OAM nanometrology by inspecting the far-field transmittance patterns of the four AM modes with a spatially distinguishable shift

To obtain the mode-sorting sensitivity on the SPPs fields, the transmittance of different total angular momentum modes (*L* = ±1, ±2, ±3, ±4) from a circular nanoaperture waveguide was numerically characterized as a function of the radius of the nanoaperture (Fig. 4a) (Methods). The results indicate that for a nanoaperture with a radius (*r*) ranging from 150 to 200 nm, the transmittance of total angular momentum modes of *L* = ±1 and ±2 is of more than two orders of magnitude larger than other higher order modes. As such, here we regard the total angular momentum mode of *L* = ± 1 as the desired one to be preferably supported by the mode-sorting nanoaperture with a radius of 150 nm. Notably, the generalized OAM nanometrology concept could be extended to sort different combinations of angular momentum modes in a sequential order, with the use of nanoring slit waveguides which exhibit single angular momentum mode sensitivity (Supplementary Fig. 5)^17,32^.

Combing the features of the spatial shift-dependent GTCs by the nanogrooves and of the mode-sorting sensitivity by nanoapertures, a physical approach for CMOS-integratable OAM nanometrology can be developed. The key steps of this approach are: (1) optical beams with different SAM and OAM modes are converted into spatially separated SPPs fields with an identical total angular momentum mode of *L* = ± 1 by multiple semi-circular nanogrooves with different GTCs; (2) the coupled SPPs fields are selectively coupled out by mode-sorting nanoapertures with *r* = 150 nm at different locations. Without loss of generality, we use four semi-circular nanogrooves with the GTC (*l*_S_) of −6, −2, +2, +6 to support the transmittance of optical beams with *l*_0_ = + 6, *s* = +1 (AM_1_), *l*_0_ = + 2, *s* = +1 (AM_2_), *l*_0_ = −2, *s* = −1 (AM_3_), *l*_0_ = −6, *s* = −1 (AM_4_), respectively.

Consequently, a CMOS-integratable OAM nanometrology device with a small footprint of 13.4 μm by 10.6 μm is fabricated on the Sb_2_Te_3_ thin film with a thickness of 100 nm (Fig. 4b). The width of the semi-circular nanogrooves is fixed as 250 nm. We numerically simulate the four-states OAM nanometrology at a visible wavelength of 561 nm. Passing the four AM beams (AM_1_, AM_2_, AM_3_, AM_4_) through the designed OAM nanometrology device leads to the spatially shifted optical transmission of the four AM beams (AM_1_, AM_2_, AM_3_, AM_4_) in the far-field region (Fig. 4c). To experimentally confirm OAM nanometrology, a quarter-wave plate (QWP) and a spatial light modulator (SLM) were used to prepare an optical beam with the determined SAM and OAM modes, respectively (Supplementary Fig. 6). As a result, optical beams with the four AM modes of AM_1_, AM_2_, AM_3_, AM_4_ are selectively coupled out by the four spatially shifted mode-sorting nanoapertures and imaged by a CMOS camera (Fig. 4d). In addition, OAM-dispersive devices composed of two shifted semi-circular nanogrooves with the GTCs of *l*_S_ = ± 2 and ± 6 were fabricated to experimentally characterize the strong OAM-dependent spatial shift (Supplementary Fig. 7). By properly defining the sensing areas for different AM modes in the detection plane of a CMOS camera^17^, our experimental results suggest a high-precision OAM nanometrology with a low modal crosstalk of < −20 dB.

## Discussion

In summary, we have demonstrated a CMOS-integratable OAM nanometrology, through the spatial engineering of plasmonic angular momentum modes in an ultrathin OAM-dispersive plasmonic topological insulator film. We have applied the outperforming plasmonic effect in an ultrathin topological insulator Sb_2_Te_3_ thin film for on-chip OAM nanometrology with an integratable CMOS camera at a visible wavelength. Notably, the current OAM nanometrology signals were imaged by a CMOS detector via a magnifying 4f optical system in the far-field region. However, owing to the recent rapid progress in nanotechnology, our demonstration holds great promise for future all-on-chip integration of the OAM nanometrology chip with a high-resolution CMOS detector^3,33^. The possibility of the direct CMOS integration is schematically illustrated in Supplementary Fig. 8. This kind of CMOS-compatible ultrathin OAM nanometrology device with a small footprint is highly compatible with other integrated devices such as vortex emitters^7^, OAM microlasers^8^, OAM manipulation metasurfaces^6,9,15^ and photodetectors^34^. The high-precision OAM nanometrology in a CMOS-compatible plasmonic topological insulator material holds a great promise for the development of ultrathin optoelectronics with versatile functionalities and ultracompact photonic integrated circuits.

## Methods

### Growth of the Sb_2_Te_3_ thin film

The 100 nm-thick Sb_2_Te_3_ thin film was grown on a glass substrate using the atomic layer deposition method. The chemical equation Sb(OC_2_H_5_)_3_ + 3[(CH_3_)_3_Si]_2_Te → Sb_2_Te_3_ + 6(CH_3_)_3_Si-OC_2_H_5_. The injection/purge times for the precursors of Sb(OC_2_H_5_)_3_ and 3[(CH_3_)_3_Si]_2_Te were 0.75 s/9 s and 0.1 s/9 s, respectively. An Ar gas booster was used on the cylinder to promote the gasification process of Sb(OC_2_H_5_)_3_ precursor. The chamber temperature was kept at 70 °C and the chamber vacuum was 2 × 10^−2^ Torr. The growth rate was 0.01 nm per cycle. X-ray diffraction (XRD) and Raman spectrum confirms that the Sb_2_Te_3_ thin films are thin film is highly crystalline and grow along the *c*-axis.

### Nanofabrication

We fabricated the ultrathin plasmonic lens and the OAM nanometrology device on a 100 nm-thick Sb_2_Te_3_ thin film using the focus ion beam (FIB) lithography method. With the use of the ion ablation, the periodic nanogrooves in the plasmonic lens and semi-circular nanogrooves and nanoapertures in the OAM nanometrology device were patterned in the Sb_2_Te_3_ thin film. In the plasmonic lens, the inner nanogroove has a radius of 5.4 μm. The groove width and the period of the nanogrooves were 250 and 605 nm, respectively. In the OAM nanometrology device, the nanoapertures were drilled through the Sb_2_Te_3_ thin film and the shallow nanogrooves were with a depth of 50 nm. The transverse shift of semi-circular nanogrooves is multiple times of the SPPs wavelength (532 nm) at a visible wavelength of 561 nm.

### Numerical simulation methods

The plasmonic lens and the OAM nanometrology in the Sb_2_Te_3_ thin film were simulated using the COMSOL Multiphysics. The optical constants of the Sb_2_Te_3_ thin film in Supplementary Figure 2 were adopted in the simulation model. A perfect-matched-layer was applied in the periphery of the computational regions to absorb the unwanted reflections. A doughnut-shaped intensity distribution, a helical wavefront, and the circular polarization were adopted in the setup of the incident beam.

Specifically, to numerically investigate the mode-sorting sensitivity by a nanoaperture, a COMSOL model was built to characterize the optical transmission of different angular momentum modes (*L*) from the Sb_2_Te_3_ thin film by sweeping the radius of the nanoaperture. This model is consisting of a shallow circular nanogroove with a thickness of 50 nm, a width of 250 nm, and an inner radius of 2 µm, as well as a drilled-through nanoaperture at the geometrical centre of the circular nanogroove with a dynamically swept radius. The grooved and ungrooved surface sides (with and without shallow nanogrooves) of the Sb_2_Te_3_ thin film were further placed onto different dielectric layers with a refractive index of 1 (air) and 1.5 (glass) and the same thickness of 1 µm, respectively. The non-diffracting OAM-carrying chiral beams (SAM) with doughnut-shaped intensity distributions are then adopted as incident beams. The SPPs are launched by illuminating the doughnut-shaped OAM-carrying chiral beams on the nanogrooves.

In addition, to numerically characterize the OAM nanometrology chip, the above COMSOL model was modified by replacing the shallow circular nanogroove with spatially displaced semi-circular nanogrooves on the Sb_2_Te_3_ thin film, wherein new nanogrooves feature a modified inner radius of 5.4 µm. Multiple drilled-through circular nanoapertures were placed at the middle location of the spatially displaced semi-circular nanogrooves with a fixed radius of 150 nm.

### Optical setup

An optical setup was used to measure the OAM-dependent spatially shifted optical signals in the far-field region. This optical system mainly consisted of a 561 nm CW-laser, a linear polarizer, an SLM for the OAM generation in the free space, a QWP for the SAM generation, and a CMOS camera. The schematic diagram is presented in Supplementary Fig. 5. The measured transmission efficiency of OAM modes was about ~2.5*10^−4^, which has enabled OAM nanometrology signals to be faithfully detected by a CMOS detector under a moderate experimental condition with the measured incident power of 1.25 µW in front of the OAM nanometrology chip.

## Electronic supplementary material

Supplementary Information

## Data Availability

All data used to obtain the conclusions in this paper are available from the corresponding author upon reasonable request.
