# Supplementary material for: Angular-momentum nanometrology in an ultrathin plasmonic topological insulator film
Source: Nat Commun. 2018 Oct 24;9:4413. doi: 10.1038/s41467-018-06952-1 (PMC6200795; doi:10.1038/s41467-018-06952-1)
Supplement: Supplementary file 1 — Supplementary Information [file 41467_2018_6952_MOESM1_ESM.pdf]

Supplementary Information

Yue, et al. "Angular-momentum nanometrology in an ultrathin plasmonic topological insulator film".

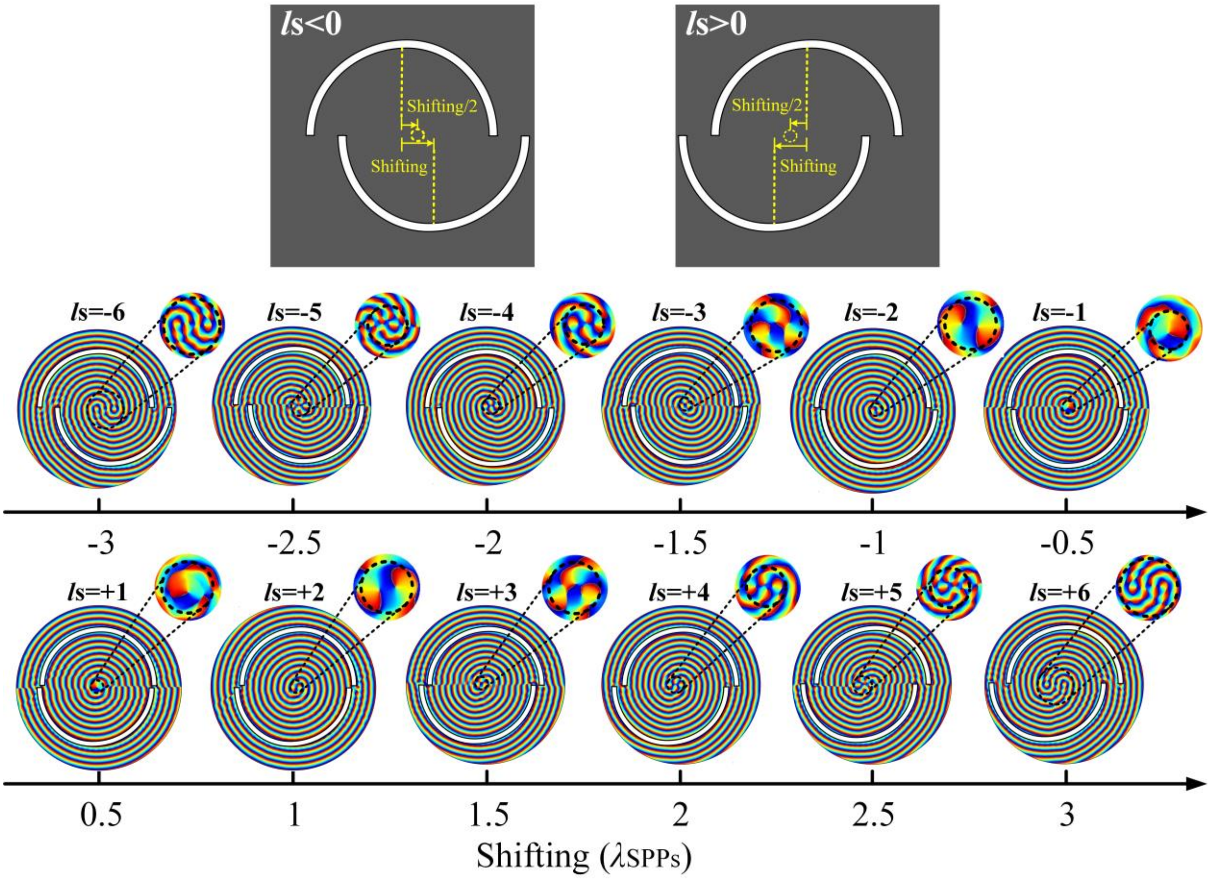

Supplementary Figure 1. Numerical characterization of the spatial shift-dependent geometric topological charges (GTCs). The insets highlight the phase distributions of different GTCs by normally illuminating an optical beam with the SAM mode of s=-1 and OAM mode of l_0=+1 on the semi-circular nanogrooves with a determined spatial shift.

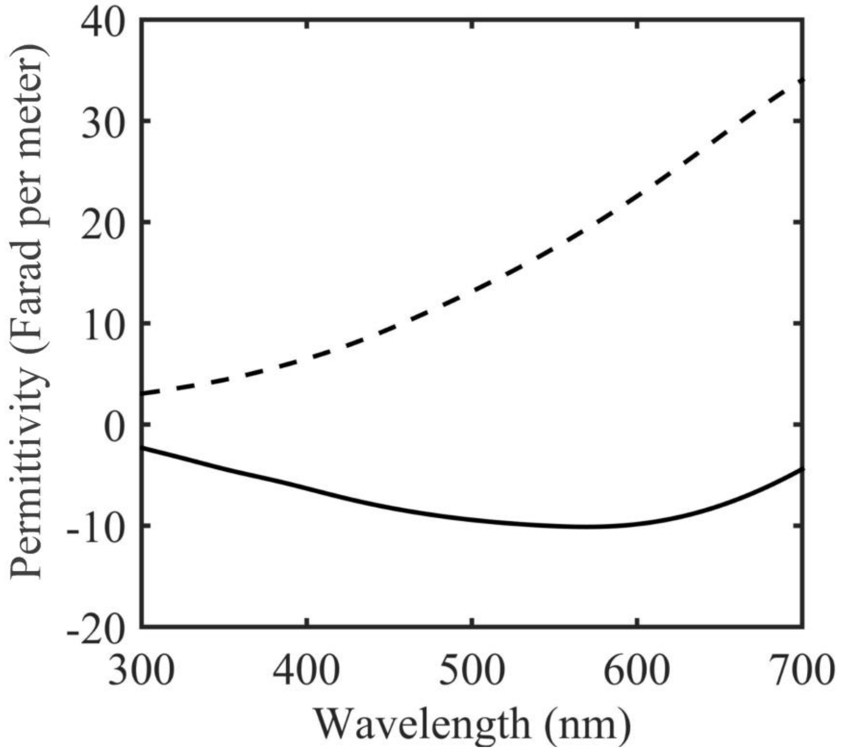

Supplementary Figure 2. Experimentally retrieved real (solid curve) and imaginary (dashed curve) parts of the permittivity spectra of the Sb_2Te_3 thin film, respectively.

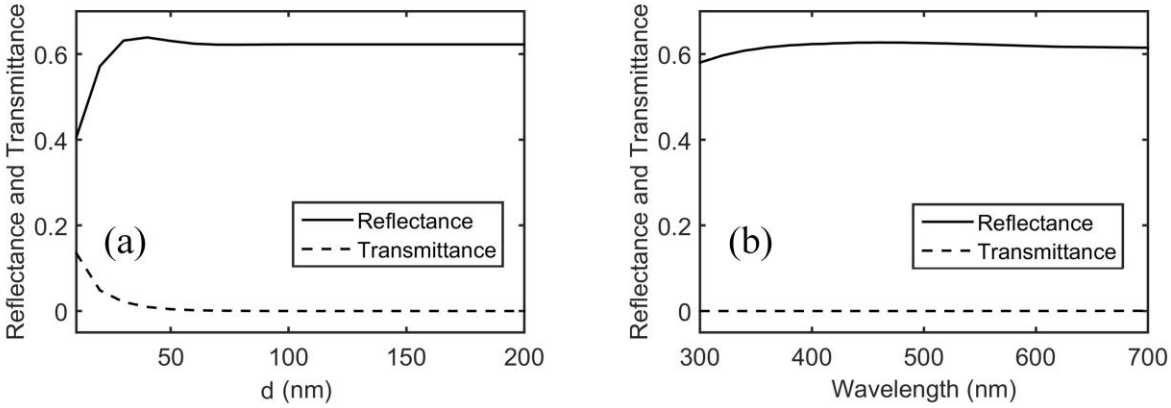

Supplementary Figure 3. Characterization of reflectance and transmittance of the Sb_2Te_3 thin film. (**a**) Simulated reflectance and transmittance spectra of the Sb_2Te_3 thin film with different thickness at the wavelength of 561 nm. (**b**) Simulated reflectance and transmittance spectra of a 100 nm-thick Sb_2Te_3 thin film as a function of the wavelengths from 300 nm to 700 nm.

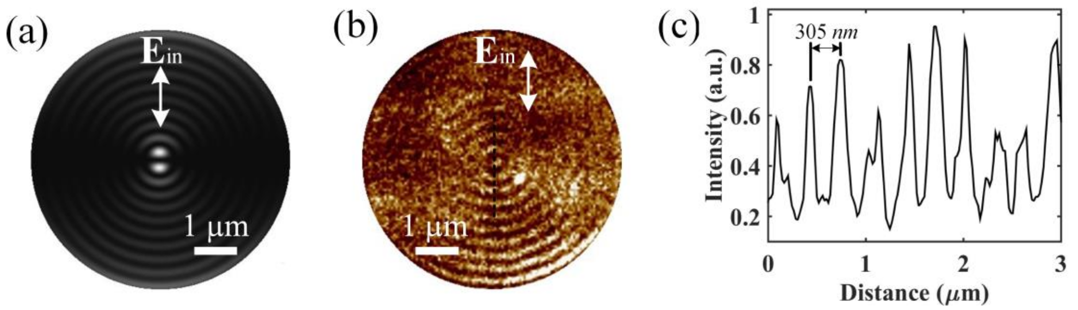

Supplementary Figure 4. Characterization of a subwavelength SPPs focus in a Sb_2Te_3 thin film. (**a**) Simulated intensity distribution of the plasmonic focus on the surface of an ultrathin plasmonic lens. The arrow shows the orientation of linearly polarized incident light. (**b**) Experimentally measured intensity distribution of the plasmonic focus by a SNOM system. (**c**) The cross-section of the measured intensity distribution of the plasmonic focus. The wavelength of the incident light beam is 640 nm. The period of the interference fringes of evanescent SPPs standing waves is around 305 nm which suggests the SPPs wavelength of 610 nm in the SNOM experiment.

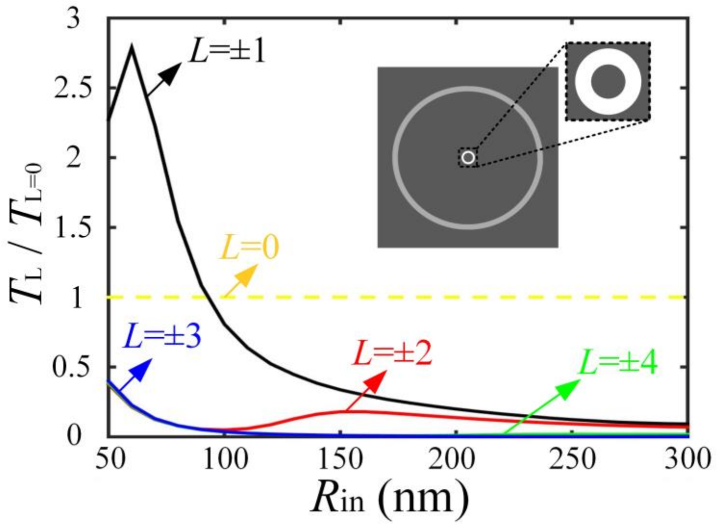

Supplementary Figure 5. Optical transmission of different total angular momentum modes *L* as a function of inner radius (*R*in) of a mode-sorting nanoring slit waveguide. The transmittance curves for different angular momentum modes are normalized by the fundamental mode result with L=0. The inset shows the schematic of the simulated nanostructures which consist of a shallow circular nanogroove and a drilled-through nanoring slit with a fixed width of 50 nm.

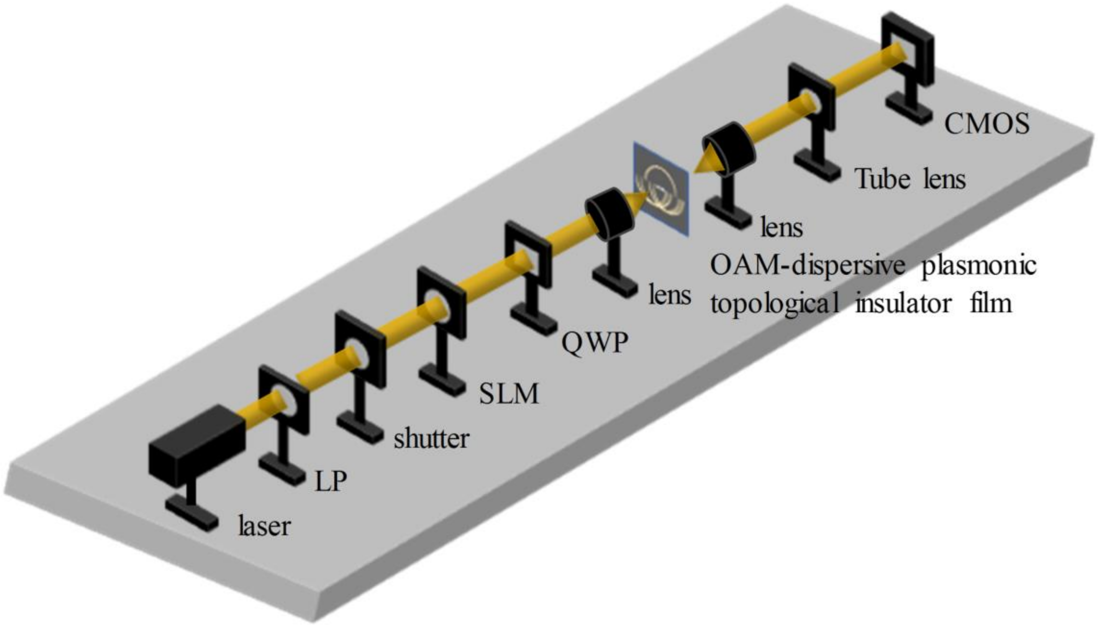

Supplementary Figure 6. Schematic drawing of the optical setup for CMOS-integratable OAM nanometrology in an ultrathin topological insulator film. LP: linear polarizer, SLM: spatial light modulator, QWP: quarter wave plate, CMOS: Complementary metal–oxide–semiconductor camera.

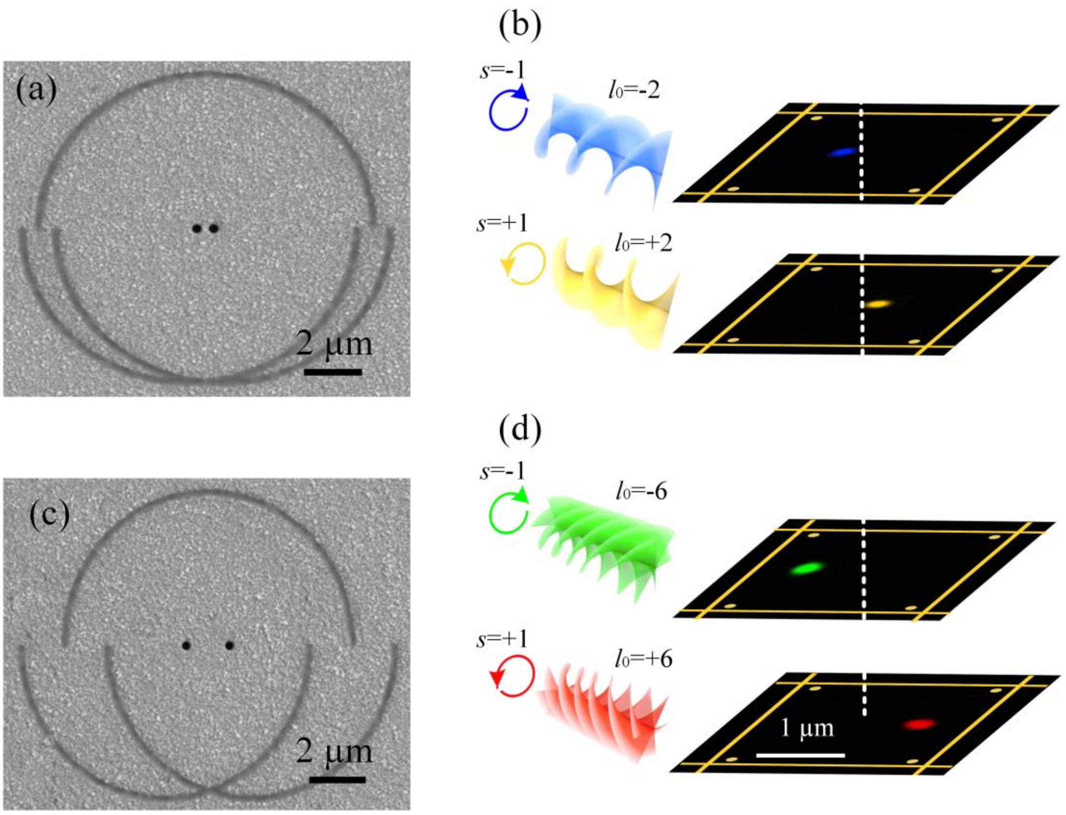

Supplementary Figure 7. Characterization of OAM-dependent spatial shift of two OAM states. (**a**) SEM image of the OAM-dispersive device with semi-circular nanogrooves with the GTCs of l_S=-2 and +2, respectively, as well as with two mode-sorting nanoapertures. (**b**) Experimental characterization of OAM-dependent spatial shift by inspecting the far-field transmittance patterns of the two AM modes of l_0=-2, s=-1 and l_0=+2, s=+1 with a spatially distinguishable shift. (**c** and **d**) The counterparts of (a and b), but with the use of nanogrooves with the GTCs of l_S=-6 and +6 to distinguish the two AM modes of l_0=-6, s=-1 and l_0=+6, s=+1, respectively.

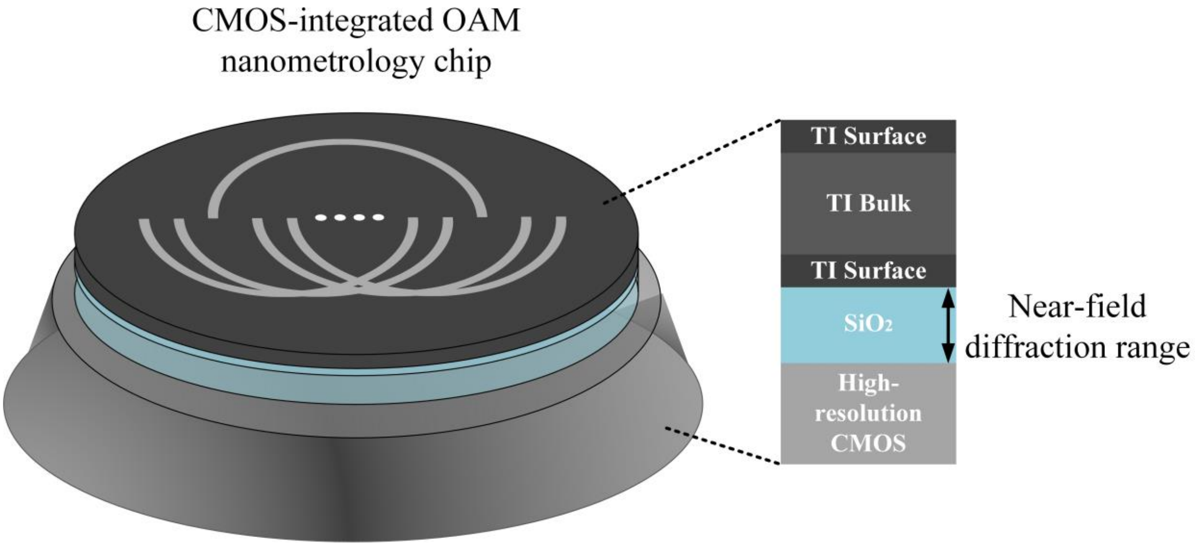

Supplementary Figure 8. An intuitive design of the CMOS-integrated OAM nanometrology chip. A high-resolution CMOS detector with sufficient sensitivity is used to directly image the OAM nanometrology signals. A transparent dielectric spacer with a subwavelength thickness is added to allow the OAM nanometrology signals from the plasmonic topological insulator thin film to be scattered out to the CMOS detector.
